# *Drosophila* Muscleblind Is Involved in *troponin T* Alternative Splicing and Apoptosis

**DOI:** 10.1371/journal.pone.0001613

**Published:** 2008-02-20

**Authors:** Marta Vicente-Crespo, Maya Pascual, Juan M. Fernandez-Costa, Amparo Garcia-Lopez, Lidón Monferrer, M. Eugenia Miranda, Lei Zhou, Ruben D. Artero

**Affiliations:** 1 Department of Genetics, University of Valencia, Valencia, Spain; 2 Department of Molecular Genetics and Microbiology Member, University of Florida Shands Cancer Center College of Medicine, University of Florida, Gainesville, Florida, United States of America; Centre de Regulació Genòmica, Spain

## Abstract

**Background:**

Muscleblind-like proteins (MBNL) have been involved in a developmental switch in the use of defined cassette exons. Such transition fails in the CTG repeat expansion disease myotonic dystrophy due, in part, to sequestration of MBNL proteins by CUG repeat RNA. Four protein isoforms (MblA-D) are coded by the unique *Drosophila muscleblind* gene.

**Methodology/Principal Findings:**

We used evolutionary, genetic and cell culture approaches to study *muscleblind* (*mbl*) function in flies. The evolutionary study showed that the MblC protein isoform was readily conserved from nematods to *Drosophila*, which suggests that it performs the most ancestral *muscleblind* functions. Overexpression of MblC in the fly eye precursors led to an externally rough eye morphology. This phenotype was used in a genetic screen to identify five dominant suppressors and 13 dominant enhancers including *Drosophila* CUG-BP1 homolog *aret*, exon junction complex components *tsunagi* and *Aly*, and pro-apoptotic genes *Traf1* and *reaper*. We further investigated Muscleblind implication in apoptosis and splicing regulation. We found missplicing of *troponin T* in *muscleblind* mutant pupae and confirmed Muscleblind ability to regulate mouse *fast skeletal muscle Troponin T* (*TnnT3*) minigene splicing in human HEK cells. MblC overexpression in the wing imaginal disc activated apoptosis in a spatially restricted manner. Bioinformatics analysis identified a conserved FKRP motif, weakly resembling a sumoylation target site, in the MblC-specific sequence. Site-directed mutagenesis of the motif revealed no change in activity of mutant MblC on *TnnT3* minigene splicing or aberrant binding to CUG repeat RNA, but altered the ability of the protein to form perinuclear aggregates and enhanced cell death-inducing activity of MblC overexpression.

**Conclusions/Significance:**

Taken together our genetic approach identify cellular processes influenced by Muscleblind function, whereas *in vivo* and cell culture experiments define *Drosophila troponin T* as a new Muscleblind target, reveal a potential involvement of MblC in programmed cell death and recognize the FKRP motif as a putative regulator of MblC function and/or subcellular location in the cell.

## Introduction

Human Muscleblind-like 1, 2 and 3 (MBNL1-3) are RNA binding proteins that have been involved in numerous diseases. Expression levels of human MBNL2 are altered in a number of cancer types [1] and MBNL1 is upregulated in schizophrenia [2] and sporadic idiopathic pulmonary arterial hypertension [3]. MBNL1 function is also impaired in myotonic dystrophy (DM), a multisystemic disease characterized by myotonia, cataracts and muscle weakness (reviewed in [4], [5]), and a fly model of spinocerebelar ataxia 8 genetically interacted with *Drosophila muscleblind* mutants [6]. In spite of their biomedical relevance, the processes in which Muscleblind proteins are required and the molecular mechanisms they use to carry out such functions are only beginning to be understood.

MBNL proteins redundantly regulate alternative splicing of *cardiac Troponin T (cTNT or TnnT2)* and *Insulin Receptor* (*IR*) minigenes in cell culture [7]. Only mouse Mbnl1, however, has been found to regulate a developmental switch from a foetal to adult-type alternative splicing pattern in a defined subset of pre-mRNAs [8]. Because Mbnl1 target RNAs are found misspliced in the human disease DM, this and additional evidences strongly support the view that reduction in MBNL1 function contributes to the DM phenotype [9]–[11]. Maintenance of a foetal splicing pattern in adult DM patients leads to misexpression of protein isoforms, or lack of them, ultimately causing the clinical symptoms [12], [13]. The origin of DM is an expansion of the trinucleotide sequence CTG in the 3′-untranslated region (UTR) of the *DMPK* gene. Mutant *DMPK* transcripts accumulate in ribonuclear foci in skeletal and cardiac muscle, fibroblast, and neuron cells of DM patients [14]–[16]. Expansion bearing mRNAs alter expression of specific genes at the level of transcription and alternative splicing [11], [17], and might also silence gene expression by RNA interference [18]. CUG repeat RNA becomes retained in nuclear foci, where it sequesters nuclear proteins including transcription factor Sp1 and alternative splicing regulators MBNL1-3 [17], [19], [20]. MBNL proteins have been shown to extensively co-localize with these ribonuclear foci *in vivo* and to bind CUG repeat-containing RNA in a repeat length dependent manner *in vitro* [14], [15], [21].

Among MBNL1 targets, regulated inclusion of exon 5 during *cTNT* pre-mRNA alternative splicing has been extensively studied in heart development. A current model proposes that a balance between the antagonistic activities of MBNL1 and polypyrimidine tract binding protein (PTB), which promote exclusion of exon 5, and CUG binding protein 1 (CUG-BP1) and embryonic lethal abnormal vision type RNA binding protein 3 (ETR-3), which promote exon 5 inclusion, instructs the spliceosome to retain or not exon 5 in mature transcripts [10]. A similar antagonism has been demonstrated in normal and DM1 myoblasts between the complex made of hnRNP H and CUG-BP1, and MBNL1, during exon 11 selection in *IR* splicing [22]. Despite their redundant activity on minigene splicing assays *in vitro*, MBNL proteins have been related with functions other than splice regulation. MBNL2 participates in the RNA-dependent localization of integrin α3 protein by binding to a specific ACACCC motif in the 3′UTR of the transcript [1]. Furthermore, MBNL3 inhibits muscle differentiation whereas *Mbnl1* knockdown mice show muscle development impairment pointing to the implication of Mbnl1 in the myogenic process [11], [23], [24].

The *Drosophila* genome contains a single *muscleblind* gene (*mbl*) that gives rise to four mature transcripts (*mblA-D*) through alternative splicing. These encode relatively small proteins that share their amino terminal region (MblA, B, C share 179 amino acids; MblD only the first 63) and differ carboxy-terminally [25]. *muscleblind* mutants die as late embryos showing disruption of sarcomeric Z bands and lack of extracellular matrix at indirect muscle attachment sites [26]. Mosaic analysis also showed that *muscleblind* was required for the terminal photoreceptor differentiation. Photoreceptors in mutant retina tissue failed to properly arrange their light harvesting structures, the rhabdomeres [25]. Muscle defects are likely caused by alternative splicing alterations in muscle transcripts of which two have been recently described in *muscleblind* mutants [27], [28]. The expression of human MBNL1 in *Drosophila muscleblind* embryos rescues embryonic lethality thus demonstrating the functional conservation between human and fly proteins [29]. Activity as alternative splicing factor in a *α-actinin* minigene splicing assay has also been shown for *Drosophila* Muscleblind, although protein isoforms were not functionally redundant and MblC showed the highest activity in this assay [27]. *muscleblind* transcript isoforms are expressed in a developmentally regulated fashion and show preferential subcellular localizations in cell culture being MblA preferentially cytoplasmatic [27].

*Muscleblind* proteins contain one (*Drosophila*) or two (vertebrate) pairs of CCCH zinc fingers [30]. Human MBNL zinc fingers have been shown to be necessary for the binding to both long CUG repeat expansions and physiological targets, whereas a C-terminal region in MBNL1 mediates homotypic interactions [31], [32]. Although several targets of MBNL proteins have been recently described [8], very little is known about the cellular processes in which MBNL proteins are implicated, their regulation or the domains required for their functions. As for *Drosophila* Muscleblind, only *α-actinin* and *CG30084* alternative splicing has been described altered in *Drosophila muscleblind* mutant embryos [27], [28] thus remaining most of *mbl* targets, and the genetic pathways in which it is involved, largely undefined. A major advantage of *Drosophila* as a model system is the ability to conduct unbiased genetic screens for enhancers and suppressors of a given phenotype *in vivo*. Here we present a genetic approach to identify new genes related to *muscleblind* function. We also used *in vivo* and cell culture techniques to understand the role of Muscleblind proteins in splicing and programmed cell death, and we define a conserved motif in MblC that impacts on the subcellular distribution of the protein.

## Results

### MblC is the most ancient isoform

To assess whether *Drosophila* Muscleblind protein isoforms were evolutionary conserved or were species-specific, we searched the genomes of organisms from representative taxonomic groups for homologous sequences. We were able to detect MblC-like protein isoforms in all species analyzed (Table 1; sequence alignments shown in Figure S1). MblC specific sequences were highly conserved in the Arthropoda group showing percentages of amino acid identity around 70% or higher. The protein was still well conserved in the Hemiptera group (pea aphid) with pairwise alignments up to 60% identical whereas this percentage went down to 40% in the Nematoda group (*C.elegans* and Ascidia). These observations suggest that an MblC-like protein appeared at least 530 million years ago when the Nematoda group and the Arthropoda lineage split.

**Table 1:** MblC-specific sequences are found in distantly related invertebrates.

	*D. melanogaster muscleblind* isoform			
	*mblA*	*mblB*	*mblC*	*mblD*
*D. simulans*	91	97	100	100
*D. yakuba*	68	99	100	-
*D. ananassae*	-	81	97	-
*D. pseudoobscura*	-	-	94	-
*D. virilis*	-	70	97	-
*D. mojavensis*	-	73	97	-
*Anopheles gambiae*	-	-	84^a^	-
*Apis mellifera*	-	-	81	-
*Bombyx mori*	-	-	68	-
*Acyrthosiphon pisum* *			56^b^	
*C. elegans*	-	-	40^c^	-
*Ascaris suum* *			49^d^	

Numbers indicate percentage of identity (best local alignment) between dynamically translated genomic DNA and isoform-specific Muscleblind protein sequences, except for species marked with an asterisk (*) for which only Expressed Sequence Tag (EST) sequences were available in databases. Accession numbers of ESTs containing isoform-specific sequences are: ^a^BM605179, ^b^BM619051.1, ^c^CV835929.1, ^d^yk381a7.5, ^e^BQ094923.1. Non-significant similarity is indicated by a minus sign (-) and data not available, at the time of the analysis, by an empty cell. Species are listed by phylogenetic distance to *Drosophila melanogaster* [65]. Multiple alignments are shown in Figure S1.

In contrast to MblC other *Drosophila* Muscleblind protein isoforms were far less well conserved. *D. simulans* is the species most closely related to *D. melanogaster* and all four Muscleblind isoforms were conserved with an identity of over 90%. MblD, however, was not detected in another closely related species belonging to the melanogaster subgroup, *D. yakuba.* This suggests that MblD appeared very recently in evolutionary terms within the *Drosophila* genus. The isoform-specific sequences of MblA and MblB were detected in the *D. yakuba* genome with identity percentages of 68 and 99% respectively, although MblB-specific sequence in *D. yakuba* was notably shorter (50 amino acid long) compared to *D melanogaster* (93 amino acid). *D. ananassae*, *virilis,* and *mojavensis* also showed an MblB-like isoform, but the conservation was almost restricted to a run of 16 consecutive alanine residues (Figure S1). These observations suggest that MblC is under strong evolutionary pressure since it has not changed significantly over at least 13 million years (almost 100% protein identity in melanogaster group species) and can be found in Nematoda. Contrarily, MblA, B and D are Muscleblind protein isoforms restricted to different melanogaster and virilis group species, probably carrying out specialized functions within these species. In summary, our data indicate that MblC isoform is the most ancient in evolutionary terms.

### MblC generates the strongest developmental defects when overexpressed

To test the functional inferences we made from the evolutionary results, we targeted Muscleblind protein isoform expression to imaginal disc tissue, muscle, photoreceptor precursors and posterior compartment within segments using the Gal4/UAS system [33] (Table S1). The strongest developmental defects, including lethality, were obtained by overexpressing *mblC*. Overexpression of *mblA* and *mblB* gave rise to far more limited defects including vein patterning defects (*T80-Gal4>UAS-mblA*) and abnormal wing position (*T80-Gal4>UAS-mblB)*, thus suggesting a more restricted developmental role. *mblD* overexpression gave no phenotype in all tissue types tested. As there was no antibody available to detect Muscleblind, we confirmed expression from the UAS transgenes by *in situ* hybridization. All transgenes were expressed to comparable levels including *UAS-mblD* ([27] and data not shown). However, neither small differences in transgene transcription (because of the qualitative nature of the experimental approach) nor differences in translation efficiency or protein turnover can be excluded. Therefore, these data suggest that MblC isoform performs important roles during *Drosophila* development, which is consistent with the broad developmental expression pattern and the high ability to rescue lethality of *muscleblind* mutant embryos that we previously described for *mblC* [27].

### A genetic screen for modifiers of a mblC overexpression phenotype

**Figure 1:**
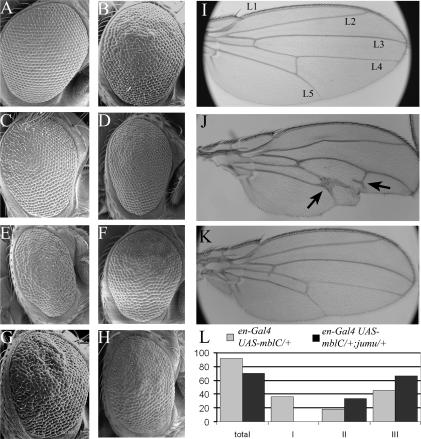
Genetic modifiers of *mblC* overexpression exhibit different interaction strengths. (A–H) Scanning electron microscopy of eyes from *Or-R* (A), *sev-Gal4 UAS-mblC/+* (B), and flies expressing *mblC* as in (B) and simultaneously heterozygous for *Traf1^GS2154^* (C), *jumu^L70^* (D), *Traf^EP578^* (E), *aret^01284^*(F), *th^5^* (G), and *nonA^f00870^* (H). Suppressors of *mblC* overexpression (C, D) ameliorated ommatidial irregularity to diverse extent. Enhancers (E–H) reduced eye size and/or increased roughness, and sometimes led to fusion of the overlying lenses. Halving *jumeaux* dose also suppressed *mblC* overexpression in the wing disc. Stereomicroscope images of wings from *Or-R* (I), *en-Gal4 UAS-mblC/+* (J), and *en-Gal4 UAS-mblC/+; jumu^L70^/+* (K). Arrows in (J) point to ectopic vein material. Bar graph representing the percentage of flies, with the genotypes indicated, that show a type I, II or III vein phenotype, or any vein phenotype (total). Phenotypic classes were defined as: I, L4 and L5 become fused and both of them disappear; II, L4 and L5 fuse but at least a vein was recognizable; III, L4 and L5 fuse, but are both recognizable. Wing in (J) would score as type III.

The mechanism by which Muscleblind proteins regulate alternative splicing is largely unknown both in *Drosophila* and vertebrates. Because *mblC* overexpression generated phenotypes amenable to genetic analysis, in particular eye morphology defects [34], and the evolutionary analysis suggested that MblC carried out critical *muscleblind* functions, we decided to perform a genetic screen for dominant modifiers of such phenotype. These modifiers provide an unbiased approximation to genes potentially involved in *muscleblind* function. *sevenless* (*sev)-Gal4 UAS-mblC* recombinant chromosomes showed a moderately rough eye phenotype (Figure 1A,B), which facilitated identification of both enhancers and suppressors. In a deficiency screen for dominant modifiers 13 out of 109 assayed deficiencies (covering around a 55% of the genome), visibly modified the phenotype being most of them dominant suppressors (Table S2). Deficiencies Df(2L)sc19-8 and Df(2L)TW137 strongly suppressed external eye morphology and were selected for further analyses. Both of them cover a wide chromosomal region including dozens of genes. By using overlapping deletions we could delimitate a candidate interacting region to 24E2; 25A5 for Df(2L)sc19-8 and to 36F1; 37B9-10 for Df(2L)TW137. Described genetic entities within these regions were individually tested for their ability to modify the *sev*-Gal4>UAS-*mblC* eye phenotype. In addition, several genes mapping within other interacting deletions, or genes known or putatively involved in RNA or DNA binding, in the DM pathogenesis or apoptosis [35], or potentially related with *muscleblind* function [6], were also tested (Table 2 and Table S3).

**Table 2:** Suppressors and enhancers of a *mblC* overexpression phenotype.

Line	M	Gene	Description	lof
*Hsp70Ab^EY01148^*	E	*Hsp70Ab*	chaperone	
*jumu^EY10708^*	E	*jumeaux*	chromatin remodelling and t.f.	*jumu^L70^* (S)
*Dp^EY09085^*	E	*Dp*	apoptosis/cell cycle regulator	*Dp^KG00660^; Dp^49Fk-1^* (E)
*amos^Roi1^; UAS-amos*	E (L)	*amos*	transcription factor	*amos^Tft-RM11^* (E)
*Aly* ^02267^	S	*Aly*	exon junction complex	
*tsu^EP567^*	E	*tsunagi*	exon junction complex	*tsu^KG04415^* (E)
*aret^01284^ aret^BG01566^*	E	*aret*	splicing factor	*aret^GS12289^* (c); *aret^PAb2^* (E)
*nonA^Df(1)^*	E	*nonA*	splicing factor	*nonA^f00870^* (E)
*rpr^GMR.PH^* (a)	E	*reaper*	pro-apoptotic	Df(3L)W4(b) (S)
*th^4^*	E	*Diap1*	apoptosis inhibitor	*th^5^* (E)
*Traf1^GS2154^*	S	*Traf1*	pro-apoptotic	*Traf1^EP578^* (E)
*tutl^GS13843^*	S	*turtle*	adhesion and motor control	*tutl^k14703;^ tutl^01085^* (E)
*CG15435^GS11154^*	S	*CG15435*	C2H2 zinc finger protein	*CG154435^KG05006^* (E)
*CG17424^NP7067^*	E	*CG17424*	stomatin-like protein (cytoskeleton)	
*CG15433^NP434^*	S	*CG15433*	acetyltransferase activity	*CG15433^KG02386^* (E)
*CG5790^NP1233^*	(L)	*CG5790*	Ser/Thr kinase	
*CG17323^GS9130^*	E	*CG17323*	UDP-glucosyltransferase	*CG17323^KG03187^* (S)
*CG31751^NP1565^.*	E	*CG31751*	aminoglycoside phosphotransferase	
*CG15625^k10217^*	E	*CG15625*	methyltransferase activity	

Modification (M) of phenotype is denoted as E (enhancer), S (suppressor) or L (lethal). lof designates other loss-of-function alleles of the modifier gene.

(a) *reaper* cDNA expressed from the glass multimer reporter (GMR) [36].

(b) Deletes all three proapoptotic genes *head involution defective*, *reaper* and *grim.*

(c) *aret^GS12289^* is a P{GS} element insertion line, which carries two UAS elements. Phenotype might be due to overexpression of nearby genes or inactivation due to insertion. t.f. signifies transcription factor.

Four out of the 14 P-element insertions mapping to 24D5; 25A2 and four of the 16 insertions mapping to 36F10; 37B-C showed interaction (Table 2). Because most P insertions tested were predicted to generate overexpression phenotypes (GS and NP lines), loss-of-function alleles of candidate genes were also checked for interaction whenever possible. All five alleles tested confirmed the interactions previously detected. Loss of function alleles of *CG15435* and *CG15433* interacted in the same direction than their corresponding NP and GS lines. *CG17323* and *turtle* loss-of-function interacted opposite to the strains initially tested. Finally, both *Traf1^EP578^* and *Traf1^GS2154^* suppressed the *mblC* overexpression phenotype.

The candidate approach identified five suppressors and 13 enhancers of the *mblC* overexpression eye phenotype (Table 2; Figure 1A–H), whereas 33 mutations did not interact including *bruno-2*, *bruno-3*, and *Diap2* alleles (Table S3). *CG5790^NP1233^* was synthetic lethal with the *sev-Gal4 UAS-mblC* chromosome, but this could be due to the additional overexpression of *mblC* driven by the NP line. For 13 of the interacting genes we tested additional alleles, which in all cases confirmed the genetic interaction. Overexpression of *Heat shock protein 70Ab* (*Hsp70Ab*), a gene encoding a protein with chaperone activity enhanced the rough eye phenotype. Four genes related to RNA metabolism, *Aly*, *tsunagi* (both components of the exon junction complex), CUG-BP1 *Drosophila* homolog *aret* (splicing and translation regulation factor) and *nonA* (splicing factor) modified *mblC* overexpression phenotype thus reinforcing previous data that implicate MblC in pre-mRNA metabolism [27]. Interestingly, several genes involved in the apoptotic process such as *Diap1*, *Traf1*, *Dp* and *reaper* strongly interacted. Reduction in the dose of proapoptotic genes *hid*, *rpr* and *grim* (Df(3L)4W) dominantly suppressed a *mblC* overexpression phenotype. *rpr^GMR.PH^* allele, which overexpresses *reaper* from a glass multimer reporter (GMR) fusion construct [36], strongly enhanced the phenotype (not shown). Crosses had actually to be made at a lower temperature (19°C) because simultaneous overexpression of *reaper* and *mblC* was larval lethal, possibly due to leaky expression in tissues other than eye imaginal discs. Consistently, halving the dose of antiapoptotic gene *thread*, the *Drosophila inhibitor of apoptosis 1* (*Diap1*) ortholog, enhanced the rough eye phenotype further suggesting that MblC overexpression sensitized cells to apoptosis. Specificity of the interaction of *mblC* overexpression with antiapoptotic genes is supported by the inability of *Diap2* dose reduction to modify the rough eye phenotype. Also *amos* and *jumeaux*, transcription and chromatin remodelling factors respectively [37]–[40], interacted with *mblC* overexpression in the eye. *jumeaux* interaction was further confirmed in wing imaginal disc tissue. Targeted *mblC* expression to posterior wing compartment by *engrailed-Gal4* originated vein fusions and lack of intervein material, affecting specially L4/L5 veins (Figure 1I-L). Simultaneous reduction of *jumeaux* dose reduced L4/L5 fusion events from 91.6 to 70.6% and also ameliorated several other aspects of the phenotype such as loss of laminar wing tissue or anterior intervein.

### Muscleblind proteins regulate *troponinT* alternative splicing

Four different loss-of-function alleles of *aret* (a Bruno protein) dominantly enhanced the *mblC* overexpression phenotype in the *Drosophila* eye. Human MBNL1 and Bruno homologs CUGBP1 and ETR3 act antagonistically on human *cTNT* transcripts to regulate inclusion of foetal exon 5 [10]. This prompted us to hypothesize that the underlying origin of the genetic interaction observed between *aret* and *muscleblind* might be conservation of their antagonism over the selection of alternative exons.

**Figure 2:**
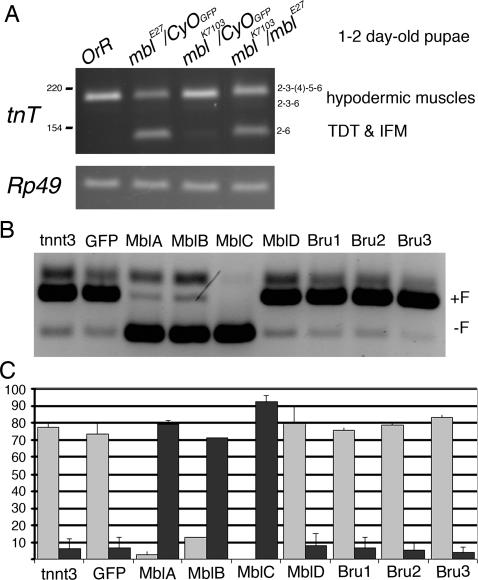
Muscleblind proteins regulate *troponin T* alternative splicing *in vivo* and in cell culture. (A) *Drosophila troponin T* splicing characterisation in wild type (*OrR*); *mbl^E27^/CyO, ubi-GFP*; *mbl^K7103^/CyO, ubi-GFP*; and the hypomorphic allelic combination *mbl^E27^/mbl^k7103^* early pupae. Molecular weight of DNA marker bands is shown on the left. Exon composition compatible with product size is shown on the right. *Rp49* is shown as control in RT-PCR. (B) Murine *TnnT3* minigene was co-transfected into HEK293T cells along with plasmids expressing *Drosophila* Muscleblind and Bruno proteins. +F indicates presence of the foetal exon and –F its absence. (C) Bar graph representing the average intensity of +F (light grey) and -F (dark grey) bands, as percentage of total, in three replica experiments, except for co-transfection of MblB that could only be amplified once. Statistically significant differences from vector alone controls (GFP lane) are denoted by an asterisk (p-value<0.01). Error bars are standard deviations. Bruno proteins did not significantly modify minigene alternative splicing.

*Mbnl1* knockout mice show splicing defects in both *cardiac Troponin T* (*Tnnt2*) and *fast skeletal muscle Troponin T* (*TnnT3*) [11]. The *Drosophila* genome presents a unique *troponin T* (*tnT*) gene, which was found to undergo tissue-specific alternative splicing during development [41]. Four splicing variants differ in the inclusion or exclusion of exons E3, E4 and E5. We performed RT-PCR amplifying exons E2 to E6 of *tnT* mRNA to detect all described splicing isoforms in *muscleblind* mutant embryo, pupae and adults. Embryo and adult RNA extractions showed no detectable differences. Early mutant pupae, however, showed an increment in the *tnT* isoform specific of tergal depressor of trochanter (TDT) and indirect flight muscles (IFM; Figure 2A) when compared to controls. The alteration was also clearly detected in *muscleblind* null heterozygotes (*mbl^E27^/CyO, ubi-GFP*), but not in hypomorphic heterozygotes (*mbl^k7103^/CyO, ubi-GFP*), thus suggesting a *muscleblind* dose effect.

Regulation of *tnT* alternative splicing by *Drosophila* Muscleblind isoforms was confirmed using an already available mouse *TnnT3* minigene whose splicing has been previously described to depend on *Mbnl1* [9]. Because they were technically more amenable than *Drosophila* S2 cells, human HEK293T cells were transiently co-transfected with the *TnnT3* minigene and plasmids expressing all four Muscleblind protein isoforms fused to GFP. We also tested the possibility that *Drosophila* Bruno proteins influenced splicing of *TnnT3* minigene transcripts by co-transfecting Bruno proteins similarly fused to GFP. The analysis showed that MblA, MblB and MblC shifted *TnnT3* splicing pattern from preferential inclusion of a foetal exon to its exclusion. Bruno proteins, however, did not significantly modify foetal exon usage (Figure 2B,C). Western blotting of HEK293T protein extracts with an anti-GFP antibody consistently failed due to protein degradation (not shown) thus precluding drawing any conclusions as for differences in splicing activity between Muscleblind protein isoforms. Titration of transfected DNA, however, showed a clear response to concentration in splicing activity, which further supports activity of Muscleblind protein isoforms in this assay (not shown).

Taken together these results demonstrate that *muscleblind* function is required for alternative splicing control of *Drosophila troponin T* mRNA and that Muscleblind protein isoforms promote the exclusion of murine *TnnT3* foetal exon from mature mRNAs in human HEK cell cultures.

### MblC overexpression activates apoptosis *in vivo*

**Figure 3:**
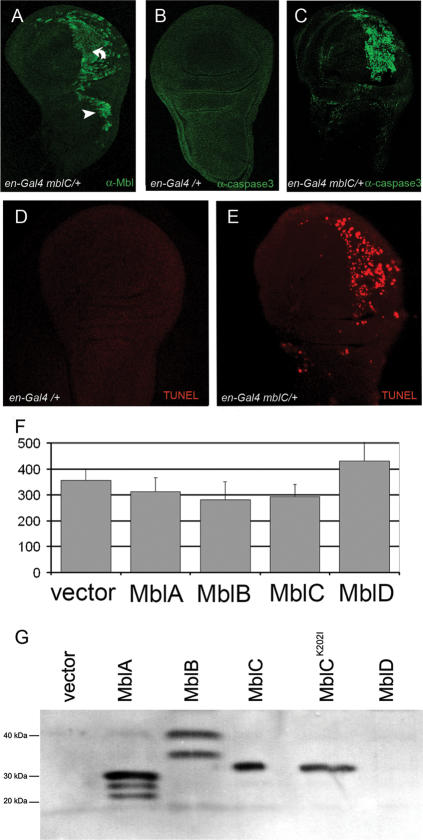
*mblC* overexpression activates apoptosis *in vivo*, but not significantly in cell culture. Confocal micrographs of third instar wing imaginal discs from *en-Gal4 mblC/+* (A,C,E) and *en-Gal4/+* controls (B,D) stained with an anti-Mbl (A), anti mammalian Caspase-3 antibody (B,C), or TUNEL assay (D,E). Wing imaginal discs of *en-Gal4 UAS-mblC* (C) flies show activation of executioner caspase-3 in cells over-expressing MblC (A) in the posterior compartment where the *en-Gal4* driver is active. Despite the fact that MblC overexpression levels are similar in posterior pouch (A, bent arrow) and notum cells (A, arrowhead), caspase-3 is not detected activated in prospective notum cells (C). A TUNEL assay to detect DNA fragmentation that results from apoptosis signalling cascades reproduced the same pattern of apoptotic cells (D,E) detected by caspase-3 activation. (F) Bar graph representing the average number (from quadruplicates) of live cells 48 h after transfection of plasmids expressing the indicated Muscleblind protein isoforms. Overexpression of Muscleblind isoforms did not significantly reduce *Drosophila* S2 cell viability in cell culture conditions. Error bars are standard deviations. (G) Western blot of protein extracts from S2 cells transfected as in (F) with the indicated Muscleblind proteins and detected with an anti-Muscleblind antibody [47]. Lower molecular weight bands in lanes MblA and MblB are degradation products. MblD could not be detected by western blotting. Predicted molecular weights are: MblA, 22.65 kDa; MblB, 34.46 kDa; MblC 26.91 kDa.

Genetic interactions with key regulators of apoptosis prompted the possibility that *muscleblind* could direct or indirectly participate in the apoptotic process. In order to confirm this possibility *in vivo*, we first analyzed the phenotype brought about by overexpression of *mblC* in the posterior compartment of the wing imaginal disc (*en-Gal4*>*UAS-mblC*). Lack of laminar tissue could originate from reduced proliferation of disc cells or from an excess of cell death (Figure 1J). Immunostaining with anti mammalian Caspase-3 antibody showed a robust activation of caspase-3 in cells overexpressing MblC under the control of the *en-Gal4* driver (Figure 3A–C), *decapentaplegic-Gal4* or *patched-Gal4* (not shown). However, not all cells overexpressing MblC showed the same susceptibility to caspase cleavage. Within the fate map of the wing imaginal disc, whereas posterior notum or ventral pleura cells did not significantly promote caspase-3 activation, posterior wing margin and pouch cells strongly activated caspase 3. In order to confirm that caspase activation was due to the activation of the apoptotic pathway, and not to other functions described for caspases (see [42] for examples), we used terminal transferase dUTP nick end labeling (TUNEL) to detect DNA fragmentation that results from apoptotic signaling cascades. Using this assay we detected several apoptotic cells in the posterior compartment of the wing disc when *en-Gal4* drove MblC overexpression. The assay also confirmed the spatially restricted susceptibility to enter apoptosis, in particular lack of apoptosis in posterior notum and pleura (Figure 3D,E).

Transfection of pro-apoptotic genes *hid*, *reaper* and *grim* into *Drosophila* S2 cells induce cell death [43]. Individual Muscleblind isoforms were similarly tested for their ability to induce apoptosis in cell culture. We first characterized endogenous expression of each *muscleblind* transcript isoform in *Drosophila* S2 cells by semiquantitative RT-PCR using isoform specific primer pairs (Figure S2). We detected predominant expression of mRNA *mblC*, similar levels of *mblA* and *mblB*, and a barely visible band for *mblD*. Next we over-expressed myc-tagged MblA-D isoforms in S2 cells and monitored cell viability by counting LacZ-expressing cells 48 h after transfection (Figure 3F,G). In these experiments we detected a reduction in the average of viable cells overexpressing MblA, MblB and MblC, whereas MblD slightly increased cell viability when compared to vector alone controls. Differences, however, were not statistically significant.

Altogether our results demonstrate that an increase of cells entering apoptosis contributes to lack of tissue in the adult wing blade in MblC-overexpressing flies and that either expression levels, or factors other than MblC, are critical to activate apoptosis *in vivo* under our experimental conditions.

### A conserved FKRP motif influences protein distribution and cell death-inducing activity of MblC

**Figure 4:**
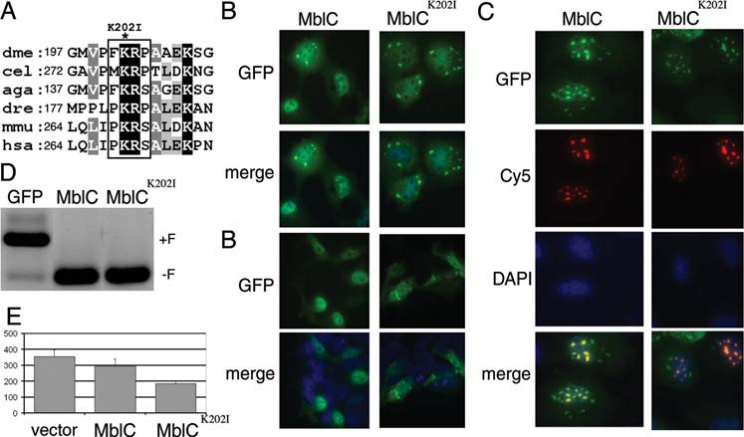
Mutation of a conserved FKRP motif reduces nuclear localization and enhances cell death-inducing activity of MblC. (A) ClustalW multiple alignment of part of *Drosophila* MblC-specific sequence (dmel) with homologous sequences from *C. elegans* (cel), *Anopheles* (aga), *Danio rerio* (dre) *mus musculus* (mmu; Mbnl1) and humans (hsa; MBNL1). SUMOplot web server predicts the *Drosophila* FKRP and *C. elegans* MKRP sequences (boxed) as sumoylation target sites. A conserved lysine (asterisk) was mutated to isoleucine in MblC^K202I^. (B) COSM6 cells transfected with 1 µg of GFP-tagged MblC protein showed preferential nuclear localization and perinuclear aggregates that increased in number in MblC^K202I^. (B) HEK293T cells transfected with 300 ng of GFP-tagged MblC showed no perinuclear aggregates whereas MblC^Κ202Ι^ still aggregated. Mutant MblC (green) co-localized with CUG ribonuclear foci (red) in the cell nucleus (blue) stained with DAPI (C). (D) *TnnT3* minigene splicing assay in HEK293T cells. GFP-tagged MblC and MblC^K202I^ promoted foetal exon exclusion to the same extent compared to transfection of the empty vector (p<0.01). (E) *Drosophila* S2 cell viability assay 48 h after transfection of normal and mutant MblC. MblC^K202I^ significantly reduced the number of viable cells (y-axis) compared to transfection of vector alone (p<0.05).

*Drosophila* Muscleblind protein isoforms sharing both their zinc fingers (MblA-C) showed different behaviour in various functional assays including differences in subcellular localization and splicing activity when over-expressed in vertebrate COSM6 cells [27]. Bioinformatics analysis of the MblC-specific sequence (64 amino acids) identified a region of conservation in distantly related Muscleblind proteins, ranging from *C. elegans* to vertebrate and human homologs (Figure 4A and Figure S1). SUMOplot, a sumoylation site prediction web server [44], identified the FKRP in *Drosophila* and MKRP in *C. elegans* as putative sumoylation sites. Small ubiquitin-related modifier (Sumo) is a 10 kDa post-translational modification that typically does not lead to protein degradation but changes in intracellular localization of proteins [45], [46]. Western blotting of protein extracts from S2 cells transfected with myc-tagged Muscleblind proteins, however, did not reveal bands of higher than predicted molecular weight (Figure 3G). Therefore, if sumoylation is actually taking place, must affect a very small proportion of the MblC protein isoform. As an alternative approach, and in order to test the relevance of the FKRP site in MblC function, we mutated lysine 201 into isoleucine by site-directed mutagenesis (Figure 4A) and tested the mutant protein (MblC^Κ202Ι^) in the functional assays we performed before in vertebrate and *Drosophila* cells with wild type MblC.

Transfection of COSM6 cells with GFP-tagged MblC^K202I^ protein showed a higher frequency of perinuclear protein foci and reduction in nuclear signal when compared to transfection of wild type MblC (Figure 4B). To check whether MblC aggregation and subcellular localization depended on the amount of transfected plasmid or were cell-type specific, we transfected HEK293T cells with a third of the constructs used initially (Figure 4B ´). No prominent perinuclear foci were observed when transfecting wild type MblC-GFP and the signal concentrated in the nucleus, but MblC^Κ202Ι^-GFP continued forming perinuclear aggregates and was also detected in the cytoplasm. We previously showed that wild type MblC co-localises *in vivo* with CUG repeat-containing ribonuclear foci [27]. Mutant MblC was similarly tested in COSM6 cells for its ability to co-localize with expanded CUG ribonuclear foci. We detected several examples of co-localization of CUG repeat RNA and MblC^K202I^-GFP in the cell nucleus and number and morphology of foci did not qualitatively differ from aggregates formed in the presence of wild type MblC (Figure 4C). Alternative splicing activity of mutant MblC was assessed using the mouse *TnnT3* minigene in HEK293T cells. In this assay MblC^K202I^ promoted foetal exon exclusion from mature transcripts to the same extent than wild type MblC (Figure 4D). Finally we tested the ability of mutant MblC to induce cell death in *Drosophila* S2 cells. Whereas MblC only marginally reduced the average number of viable cells upon transfection, reduction of cell viability 48 h after MblC^K202I^ overexpression was to approximately 52 % of control and statistically significant (Figure 4E).

Taken together these results identify the FKRP motif in MblC as a putative site for post-translational modification. They also show that the FKRP sequence influenced subcellular localization of the protein and that mutation of the motif enhances the cell death inducing activity of MblC overexpression in cell culture.

## Discussion

Using *Drosophila* as a model organism, here we report the first screen specifically addressed to identify gene functions related to the biomedically important protein Muscleblind. In support of the relevance of our results, we show the strong functional conservation between fly and vertebrate Muscleblind proteins. Furthermore, we generated data supporting that Muscleblind can induce apoptosis *in vivo* in imaginal disc tissue and identified a conserved motif in the MblC protein isoform that conferred pro-apoptotic activity in *Drosophila* cell culture when mutated. Noteworthy, this is the first conserved motif (besides CCCH zinc fingers) that is associated with a particular function in Muscleblind proteins.

### MblC-specific sequences, but not other isoforms, are detected in several protostomes and human MBNL1

Whereas most vertebrates include three *muscleblind* paralogues in their genomes, a single *muscleblind* gene carries out all *muscleblind*-related functions in *Drosophila*. These functions are probably accomplished through alternative splicing, which generates four Muscleblind protein isoforms with different carboxy-terminal regions. We performed an evolutionary analysis with isoform-specific protein sequences in order to assess conservation of alternative splicing within protostomes. We detected MblC-like isoforms even in the nematodes *C. elegans* and *Ascaris suum* but not MblA, B or D, that were only consistently found within Drosophilidae. Interestingly, also vertebrate *Mbnl1* genes included MblC-like sequences (Figure 4A). This finding, together with previous studies where we reported that *mblC* was the isoform with the strongest activity in a *muscleblind* mutant rescue experiment and *α-actinin* minigene splicing assay [27] point to *mblC* as the isoform performing most of *muscleblind* functions in the fly. Despite this Muscleblind isoforms are partially redundant. Both *mblA* and *B* partially rescued the embryonic lethality of *muscleblind* mutant embryos [27] and were able to similarly promote foetal exon exclusion in murine *TnnT3* minigene splicing assays. MblD showed no activity in splicing assays or *in vivo* overexpression experiments. However, we show a marginal increase in cell viability in cell death assays. Using isoform-specific RNAi constructs we plan to re-evaluate the function of Muscleblind isoforms both *in vivo* and in cell culture.

### A genetic screen involves Muscleblind in defined cellular processes

Although the regulation of alternative splicing by Muscleblind proteins is an established fact, the cellular processes in which the protein participates are largely unknown. Genetic screens provide a way to approach those processes as they interrogate a biological system as a whole. Overexpression of MblC in the *Drosophila* eye originated an externally rough eye phenotype that was temperature sensitive, thus indicating that was sensitized to the *muscleblind* dose. We performed a deficiency screen and tested several candidate mutations for dominant modification of the phenotype. We identified 19 genes of which more that half can be broadly classified as involved in apoptosis regulation (*rpr*, *th* and *Traf1*), RNA metabolism (*Aly*, *tsu*, *aret* and *nonA*) or transcription regulation (*jumu*, *amos, Dp*, *CG15435* and *CG15433*), whereas the rest do not easily fall into defined classes. *muscleblind* has been shown to regulate *α-actinin* and *troponinT* alternative splicing both *in vivo* and in cell culture ([27], [28]; this work). The genetic interaction with the *Drosophila* homolog of human splicing factor CUG-BP1 (*aret*) and *nonA* supports a functional relationship in flies. The antagonism between MBNL1 and CUG-BP1 has actually been shown in humans [10], whereas RNA-binding protein NonA might be relevant to Muscleblind sequestration by CUG repeat RNA in flies [47].

Reduction of dose of exon junction complex (EJC) components *tsunagi* and *Aly* also modified MblC overexpression phenotype. EJC provides a binding platform for factors involved in mRNA splicing, export and non-sense mediated decay (NMD). This suggests a previously unforeseen relationship between Muscleblind and EJC, perhaps helping to couple splicing to mRNA export. Consistently, *Aly* mutations enhanced a CUG repeat RNA phenotype in the *Drosophila* eye [35]. A similar coupling between transcription and splicing might explain the identification of a number of transcription factors in our screen. Of these, we studied in some detail the effect of *jumu* alleles in the eye and wing MblC overexpression phenotypes. Loss of function *jumu* mutations suppressed both wing defects and rough eye, whereas had no effect on unrelated overexpression phenotypes (not shown) thus suggesting that the interaction was specific.

Mutations in the *Drosophila* homolog of vertebrate *Inhibitor of Apoptosis* (*Diap1* or *thread*) dominantly enhanced the rough eye phenotype. Consistently with the specificity of the interaction, a second *Drosophila* paralog, *Diap2*, did not interact. Also, a deficiency that removes the *Drosophila* proapoptotic genes *hid*, *reaper* and *grim* (which inhibit *thread*) was a dominant suppressor while *reaper* overexpression in eye disc enhanced the phenotype. Interestingly the human homolog of *Drosophila* Hsp70Ab, Hsp70, has been related to apoptosis as it directly interacts with Apaf-1 and Apoptosis Inducing Factor (AIF) resulting in the inhibition of caspase-dependent and caspase-independent apoptosis [48]. All these genetic data are consistent with MblC overexpressing eye discs being sensitized to enter apoptosis, although we did not detect increase in caspase-3 activation in third instar eye imaginal disc overexpressing MblC (not shown).

### Do Bruno proteins antagonize Muscleblind activity in flies?

Human MBNL1 and CUB-BP1 cooperate to regulate the splicing of *cardiac TroponinT* (*cTNT*, [7]). We detected splicing defects in *Drosophila troponinT* mRNA in *muscleblind* mutant pupae. Interestingly, we detected an abnormal exclusion of exon 3 in *muscleblind* mutant pupae, encoding a glutamic acid-rich domain homologous to the foetal exon of *cTNT* regulated by human MBNL1 [41]. *Drosophila* exon 3 is only absent in the *troponinT* isoform expressed in TDT and IFM muscles and probably confers specific functional properties much like the foetal exon does in humans [49]. This identifies *troponinT* as a new target of Muscleblind activity in flies.

CUG-BP1 protein has been described to antagonize MBNL1 exon choice activity in *IR* and *cTNT* pre-mRNAs. Moreover, we detected a genetic interaction between MblC overexpression and *aret* loss of function mutations. In order to further characterize the functional interaction between Muscleblind and Bruno proteins we checked their ability to regulate murine *TnnT3* in human cell culture. MblA, B and C showed strong activity on *TnnT3* mRNA but no significant activity was detected for any Bruno protein. This shows a strong functional conservation between fly and vertebrate Muscleblind proteins as *Drosophila* isoforms can act over a murine target in a human environment. In contrast, Bruno proteins might not conserve the regulatory activity over *troponinT* mRNA described for their vertebrate homologues or at least they were not functional in the cellular environment used in this assay. Because GFP-tagged Bruno proteins were only weakly expressed in HEK cells under our experimental conditions, the level of expression might be insufficient to overcome endogenous Muscleblind activity in cell culture. Furthermore, Bruno proteins might antagonize Muscleblind on a different subset of RNA targets. Although *bruno1* has been shown to regulate splicing of some transcripts in S2 cell culture [50] and Bruno3 binds the same EDEN sequence than human CUG-BP [51], no *in vivo* experiments have addressed the functional conservation between fly and vertebrate Brunos. Bruno1 is expressed in the germ line [52] where it acts as translational repressor of *oskar* and *gurken* mRNAs [53], [54].

### Muscleblind overexpression activates apoptosis

Wing imaginal discs stained with anti-caspase-3 and with TUNEL showed that activation of apoptosis was not general in cells expressing MblC but restricted to defined regions within the disc, in particular the wing blade. The spatial constraints that we observed within the imaginal disc might explain the small effect detected when expressing Muscleblind proteins in S2 cells. MblC might require the presence of other factors to be able to unleash programmed cell death. Alternatively, the level of overexpression may be critical and transfected Muscleblind proteins may not reach a critical threshold in *Drosophila* S2 cells. MblC activation of apoptosis could reveal a direct regulation of apoptotic genes at RNA level or be an indirect effect. Several apoptotic genes produce pro-apoptotic or anti-apoptotic isoforms depending on the regulation of their alternative splicing [55]. MblC could be similarly regulating protein isoforms originating from one or a number of key apoptotic genes at the level of pre-mRNA splicing. Alternatively, MblC could be regulating isoform ratio of a molecule indirectly related to programmed cell death, for example a cell adhesion molecule causing apoptosis by inefficient cell attachment to the substrate. Furthermore, human MBNL proteins are implicated not only in splicing but also in RNA localization [1], [7], a process that if conserved in flies can potentially impinge in apoptosis regulation.

The analysis of MblC-specific sequence revealed a region conserved in Muscleblind proteins from nematodes to humans. Post-translational prediction programs found a motif (FKRP) weakly resembling a sumoylation target site. However, our results in S2 cells suggest that sumoylation, if actually taking place, modifies only a small fraction of MblC proteins. FKRP may alternatively participate in an interaction with a Muscleblind partner potentially regulating activity or location in cell compartments, assist in protein dimerization [32], or others functions. We mutated the FKRP site and performed a number of functional assays using the mutant MblC. Whereas MblC^K202I^ excluded foetal exon in *TnnT3* minigene splicing assays and bound CUG repeat RNA like its wild type counterpart, the mutant protein showed a different preferential distribution in human cells and significantly increased cell death activation upon overexpression. The mechanism by which the FKRP site influences subcellular distribution and cell death-inducing activities is currently unknown, but nevertheless constitutes the first motif, other than zinc fingers, that is associated with a function within Muscleblind proteins.

## Materials and Methods

### Evolutionary study of Muscleblind isoforms

The tBLASTn algorithm [56] was used to search isoform-specific Muscleblind protein sequences in insect and *C. elegans* genome assembly DNA and Genbank EST databases using the BLAST tools posted in Flybase [57] and Wormbase [58], respectively. *Acyrthosiphon pisum* ESTs searches (tBLASTn) were performed using the aphidbase (www.aphidbase.org). *Ascaris suum* EST searches (tBLASTn) were performed using the corresponding Wellcome Trust Sanger Institute project (www.sanger.ac.uk/) and the University of Edinburgh nematode database (www.nematodes.org). Searches were performed without filtering for low complexity sequences.

### *Drosophila* genetics and phenotypic analysis

The strong hypomorph, possibly null, allele *mbl^E27^* was reported in [25], whereas the weak allele *mbl^k7103^* was in [59]. *UAS-mblA* and *UAS-mblC* flies are described in [34], and *UAS-mblB* in [27]. To generate *UAS-mblD* transgenic flies, a 2 kb *EcoR*I fragment from *mblD* cDNA [25], including the putative open reading frame, was released from pBluescript by *Not*I/*EcoR*I digestion and subcloned into pUAST opened up with *Xho*I/*Not*I. The transgene was microinjected into *yw* flies following standard methods for *Drosophila* germ line transformation.

Expression under the control of the *sev* enhancer in R3/R4, R1/R6 and ectopically in R7, the mystery and cone cells was achieved with the *sev-Gal4* driver (gift from M. Mlodzik; Mount Sinai School of Medicine, New York), and in the posterior compartment of every segment with the *en-Gal4*. Transgenic lines A1 and E1 of *UAS-mblC*, and Gal4 drivers *sev-Gal4* and *en-Gal4*, were respectively combined by meiotic recombination using standard procedures. Recombinant chromosomes *sev-Gal4 UAS-mblC(A1).1* and *en-Gal4 UAS-mblC(E1).1* were subsequently used in this work. Repositories *Drosophila Genomics Resource Center* (DGRC), Bloomington *Drosophila* Stock Center (BDSC), and Kyoto fly were used to obtain the deficiency kit (BDSC), mutant stocks and *en-Gal4* driver. All P-element insertion lines screened were GS (two UAS sequences in opposite directions [60]) or NP (Gal4 enhancer trap; [61]). *sev-Gal4 UAS-mblC(A1).1/TM3* females were crossed to deficiency or P-element carrying males. All crosses were maintained at 25^a^C except for the interaction with *Gal4-rpr* that was at 19°C. *Oregon-R* (*Or-R*) was used as reference strain.

For phenotypic analysis flies were incubated in SH buffer (25% glycerol, 75% ethanol) overnight. After a rinse with water, wings were dissected out in water and mounted in Faure's mounting media [62]. For quantification, wing phenotypes of F1 flies with the genotypes *en-Gal4 UAS-mblC(E1).1/+* (controls; n = 12) and *en-Gal4 UAS-mblC(E1).1/+; jumu^L70^/+* (n = 17) were scored according to the type and degree of alteration observed in the posterior wing margin (lack of tissue and ectopic vein material). For scanning electron microscopy adult flies were treated as described in [63].

### Immunohistochemistry and Terminal transferase dUTP Nick End Labeling (TUNEL) assay

For immunodetection of executioner caspases in *Drosophila* we used an anti mammalian caspase-3 active polyclonal antibody from R&D systems (AF835). Third instar wing discs were dissected out in cold PBS, fixed for 30 min at room temperature in a 1:1 mixture of 8% formaldehyde: PEM 2× (0.2 M PIPES, 2 mM MgCl_2_, 2 mM EGTA) and permeabilized in PBS + Triton 0.3% (PBT) at room temperature (3 washes, 20 min each). Following blocking in PBT + 10% goat serum, discs were incubated with a 1∶100 dilution of primary antibody for 2 h at room temperature or overnight at 4°C. After 8 washes with PBT (15 min each), discs were incubated for 1–2 h with anti-rabbit secondary antibody conjugated to biotin at a 1∶200 dilution. Secondary was washed 8 times with PBT (15 min each) and incubated with streptavidin-FITC (1∶200) for 30 min. After three washes (20 min each), discs were postfixed for 30–60 min in fixation mixture as above. Fixative was washed 3 times with PBT and discs were mounted in mounting media (Dako Cytomation). All steps were with gentle agitation (nutator mixer).

TUNEL labelling was performed using an *in situ* cell death detection kit (Roche) adapted to *Drosophila* imaginal disc tissue according to [64]. Briefly, third instar larvae were dissected in PBS and fixed in 0.1 M PIPES, pH6.9, 1 mM EDTA, 1% Triton X-100, 2 mM MgSO_4_, 1% formaldehyde for 30 min. Samples were then washed three times in PBS+0.1% Triton X-100, twice in PBS+0.5% Triton (10 min each) and transferred to permeabiliation solution (sodium citrate 0.1 M in PBT) for 30 min at 65°C. Discs were washed twice in 5× PBT (10 min each), rinsed three times in 1× PBT, and incubated in 50, 50 and 45 µl of labelling solution for 10 and 20 min (at room temperature), and 10 min (at 37°C), respectively. Terminal transferase (enzyme solution) was added to a concentration of 1× and samples incubated at 37°C for 3 h. Reaction was stopped by transferring discs to 5× PBT and three rinses in 1× PBT. Images were taken on a Leica TCS SP confocal microscope.

### Constructs

*bruno* open reading frames were amplified by high fidelity PCR (TripleMaster PCR system, Eppendorf) from cDNAs LD29068 (*aret/bruno1*), LD19052 (*bruno2*) and LD31834 (*bruno3*) using primers carrying adaptors for enzymatic restriction (Table S4) and cloned into pGFP-N3 linearized with the same enzymes. MblC-specific FKRP site was mutated by oligonucleotide-directed mutagenesis. MblCK202I and MblC-PstI primers were used to amplify a fragment of MblC, which substituted A by a T that changed Lys202 into Ile202. For convenience, the MblCK202I primer also introduced a silent *Pst*I restriction site that facilitated recognition of mutated construct. PCR product and vector pEGFP-N3 were digested with *BstX*I y *Sal*I and ligated in a standard reaction. For cell culture experiments, myc-tagged *muscleblind* constructs [27] were *Bgl*II and *Not*I digested from pMV vector and cloned into the *Drosophila* expression vector pIEI4 opened up by *BamH*I and *Not*I digestion. All constructs were confirmed by sequencing.

### Cell culture assays and western blotting

Minigene splicing assays were carried out in human HEK293T cells as previously described [27] using 0.5 µg of a mouse *TnnT3*minigene [9] and 0.25 µg and 0.5 µg of pEGFP-N3 (Clontech) plasmids expressing Muscleblind or Bruno proteins fused to GFP (see [27] and above for a description of these constructs) using 1 ml of Lipofectamine in presence of Optimem Media. COSM6 cells were transfected with 1 µg of plasmids expressing GFP-tagged Muscleblind proteins and 1 µg of carrier DNA (pSP72) for subcellular localization studies, or 1 µg of plasmid expressing (CUG)_197_ plus 1 µg of plasmid expressing GFP-tagged Muscleblind proteins for colocalization assays. HEK293T cells were transfected with 300 ng of plasmids carrying the Muscleblind:GFP fusion. Detection was carried out as described previously [27]. For cell death assay S2 cells were transfected and treated as described [43] using 0.9 µg of plasmids expressing Myc-tagged Muscleblind isoforms [27].

Total protein extracts from S2 *Drosophila* cells were performed 24 h after transfection by resuspending cells in loading buffer. Samples were denatured for 5 min at 100°C, electrophoresed on 12% PAGE-SDS gels, transferred to PVDF membranes and immunodetected following standard procedures. Primary anti-Muscleblind antibody, with the capacity to recognize all four protein isoforms [47], was used at 1:5000 in blocking solution (3% BSA). Chemiluminiscent detection was with the ECL substrate (Pierce) following recommendations by the provider.

### Reverse Transcriptase Polymerase Chain Reaction (RT-PCR) analysis of alternative splicing

Individuals with the *mbl^E27^/mbl^k7103^* genotype were obtained by crossing *mbl^E27^*/*CyO; ubiquitous-GFP* and *mbl^k7103^*/*CyO; ubiquitous-GFP* flies and identified by the lack of fluorescence using a GFP fluorescence module mounted on a Leica MZ APO stereo microscope. 12 to 24 h after egg laying embryos, early pupae (1–2 days after pupation) and adults (6 h after emergence from puparium or older) were collected to analyze *tnT* splicing pattern.

Total RNA from flies or minigene splicing assays in cell culture was extracted using Tri-reagent (Sigma) and treated with DNase I (Invitrogen or Roche). Reverse transcription (RT) was performed using 1 µg (flies) or 5 µg (cell cultures) of total RNA, Superscript II RNase H- and random hexamers following instructions from the provider (Invitrogen). 1 µl of cDNA was used in a standard 20 µl PCR reaction with *Thermus thermophilus* DNA polymerase (Netzyme, NEED) and primers TNTE2 and TNTE6 for fly samples, 1938/1956 for minigene assays, or isoform-specific primer pairs to amplify *muscleblind* isoforms (Table S4). Cycling conditions were 94°C for 2 min and 25 cycles of 94°C for 30 s, 56°C for 30 s, and 72°C for 30 s. 1 µl of a 1∶100 dilution of cDNA was used in a similar 25 cycle PCR with primers Rp49 for/rev as control for reverse transcriptase reaction and RNA input. Rp49 primers were designed encompassing an intronic region to detect contamination by genomic DNA. PCR products were resolved in 2% agarose gels. 1D-Manager software (TDIsa) was used to quantify gel images. The presence of the foetal exon of *TnnT3* in the PCR products was confirmed by sequencing.

## Supporting Information

Figure S1 Clustal W multiple sequence alignment of isoform-specific Muscleblind sequences. Clustal W (1.82) multiple sequence alignment of evolutionarily conserved Muscleblind protein isoforms. Coordinates refer to the isoform-specific sequence. The FKRP site in the MblC specific sequence is highlighted in grey. Sequence names include the Muscleblind isoform and “x” to indicate isoform-specific sequence, followed by the genus (first letter) and species (first two letters). Species analyzed are listed in Table 1.
(9.92 MB TIF)

Figure S2 Drosophila S2 cells express mblA, B, C and D mature transcripts to different levels. A semiquantitative RT-PCR amplified isoform-specific regions from two independent RNA samples. Rp49 is shown as control.
(2.70 MB TIF)

Table S1 Targeted expression of mbl transcript isoforms originates markedly different morphological phenotypes. Gal4 lines used drive expression ubiquitously to imaginal (T80-Gal4) or muscular tissue (Mhc-Gal4), to specific eye cell types (sev-Gal4) or to the posterior compartment of segments (en-Gal4).
(0.03 MB DOC)

Table S2 Interacting deficiencies. Deficiencies that dominantly modified the mblC eye overexpression phenotype. S denotes suppression, E enhancement and -no interaction. Number of + signs qualitatively indicate the strength of the phenotypic modification. (*) Genetic data (Flybase) indicate that region from approximately 24D2 to 24E2 is actually present in the deletion. Cytogenetic data is according to Flybase.
(0.03 MB DOC)

Table S3 Non-interacting genes. Alleles tested for genetic interaction with a mblC overexpression phenotype that did not interact.
(0.05 MB DOC)

Table S4 Primer sequences used in this study. Names of primers used in the generation of GFP-tagged Bruno proteins include the first four characters of their cDNA names and the restriction site introduced. MblCK202I and MblC-PstI were used to perform site-directed mutagenesis on MblC-specific motif FKRP. TNTE2 and TNTE6 were used to amplify Drosophila troponin T transcripts. Rp49f and Rp49r amplify Rp49 mRNA as control for reverse transcriptase efficiency and RNA input.
(0.04 MB DOC)
