# Supplementary figures and images for: Drosophila Muscleblind Is Involved in troponin T Alternative Splicing and Apoptosis
Source: PLoS One. 2008 Feb 20;3(2):e1613. doi: 10.1371/journal.pone.0001613 (PMC2238819; doi:10.1371/journal.pone.0001613)

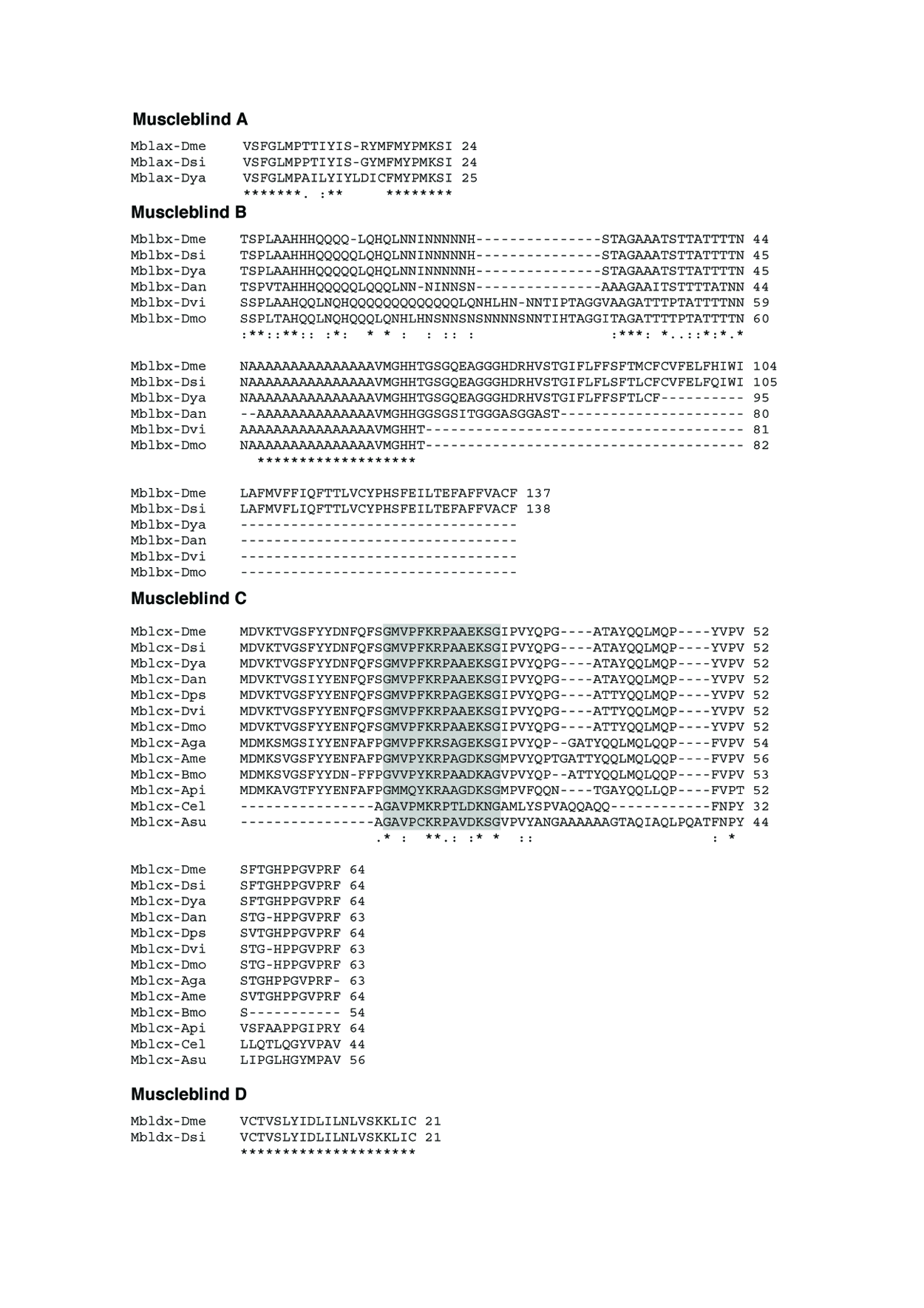

Supplement: Figure S1 — Clustal W multiple sequence alignment of isoform-specific Muscleblind sequences. Clustal W (1.82) multiple sequence alignment of evolutionarily conserved Muscleblind protein isoforms. Coordinates refer to the isoform-specific sequence. The FKRP site in the MblC specific sequence is highlighted in grey. Sequence names include the Muscleblind isoform and “x” to indicate isoform-specific sequence, followed by the genus (first letter) and species (first two letters). Species analyzed are listed in Table 1. (9.92 MB TIF) [file pone.0001613.s001.tif]

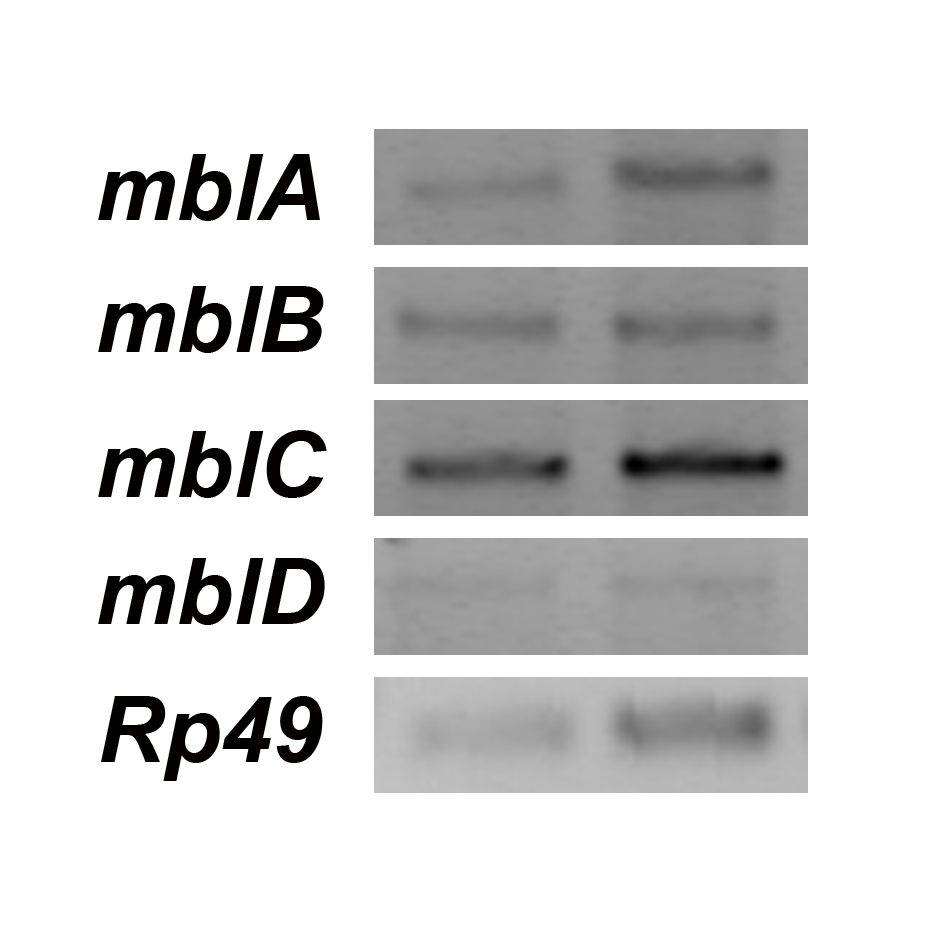

Supplement: Figure S2 — Drosophila S2 cells express mblA, B, C and D mature transcripts to different levels. A semiquantitative RT-PCR amplified isoform-specific regions from two independent RNA samples. Rp49 is shown as control. (2.70 MB TIF) [file pone.0001613.s002.tif]
