# Supplementary material for: Drosophila Muscleblind Is Involved in troponin T Alternative Splicing and Apoptosis
Source: PLoS One. 2008 Feb 20;3(2):e1613. doi: 10.1371/journal.pone.0001613 (PMC2238819; doi:10.1371/journal.pone.0001613)
Supplement: Table S1 — Targeted expression of mbl transcript isoforms originates markedly different morphological phenotypes. Gal4 lines used drive expression ubiquitously to imaginal (T80-Gal4) or muscular tissue (Mhc-Gal4), to specific eye cell types (sev-Gal4) or to the posterior compartment of segments (en-Gal4). (0.03 MB DOC) [file pone.0001613.s003.doc]

| ***UAS*** | ***T80-Gal4*** | ***Mhc-Gal4*** | ***sev-Gal 4*** | ***en-Gal4*** |
| --- | --- | --- | --- | --- |
| ***mblA*** | Vein defects  Flightless | Flightless  Wing position  defects | Photoreceptor  defects | Vein and  bristle defects |
| ***mblB*** | Flightless  Wing position  defects | No phenotype | No phenotype | No phenotype |
| ***mblC*** | Severe vein and  bristle defects.  Folded wings,  lethality | Flightless  Wing position  defects | Rough eye.  Planar polarity  defects and lack  of ommatidia | Lack of  laminar  tissue and  vein defects |
| ***mblD*** | No phenotype | No phenotype | No phenotype | No phenotype |
