# Supplementary material for: Drosophila Muscleblind Is Involved in troponin T Alternative Splicing and Apoptosis
Source: PLoS One. 2008 Feb 20;3(2):e1613. doi: 10.1371/journal.pone.0001613 (PMC2238819; doi:10.1371/journal.pone.0001613)
Supplement: Table S2 — Interacting deficiencies. Deficiencies that dominantly modified the mblC eye overexpression phenotype. S denotes suppression, E enhancement and -no interaction. Number of + signs qualitatively indicate the strength of the phenotypic modification. (*) Genetic data (Flybase) indicate that region from approximately 24D2 to 24E2 is actually present in the deletion. Cytogenetic data is according to Flybase. (0.03 MB DOC) [file pone.0001613.s004.doc]

| **Deficiency** | **Modification** | **Cytogenetic breakpoints** |
| --- | --- | --- |
| ***Df(2R)017*** | S*^+ +^* | 56F5; F15 |
| ***Df(2L)C144*** | S*^+ +^* | 23A1-2; 23C3-5 |
| ***Df(2L)sc19-8*** ^(*)^  *Df(2L)ed1*  *Df(2L)sc19-4* | S*^+ + +^*  E  - | 24C2-8; 25C8-9  24A2; 24D4  25A5; 25E5 |
| ***Df(2L)TW137***  *Df(2L)H20*  *Df(2L)M36F-S6* | S*^+ + +^*  E^+ +^  - | 36C2-4; 37B9-10  36A8-9; 36F1  36F2-6; 36F2-6 |
| ***Df(3R)TpI10*** | S*^+ +^* | 83C1-2; 84B1-2 |
| ***Df(3L)66C-G28*** | S*^+ +^* | 66B8-9; 66C9-10 |
| ***Df(3L)vin5*** | S*^+ +^* | 68A2-3; 69A1-3 |
| ***Df(3L)BSC12*** | S*^+ +^* | 69F6-70A1; 70A1-2 |
| ***Df(3L)st-f13*** | S*^+ +^* | 72C1-D1; 73A3-4 |
| ***Df(3L)XS-533*** | S*^+ +^* | 76B4; 77B |
| ***Df(2R)w45-30n*** | E*^+ + +^* | 45A6-7; 45E2-3 |
| ***Df(2R)CX1*** | E*^+ +^* | 49C1-4; 50C23-D2 |
| ***Df(2L)r10*** | E*^+ +^* | 35D1; 36A6-7 |
