# Supplementary material for: Drosophila Muscleblind Is Involved in troponin T Alternative Splicing and Apoptosis
Source: PLoS One. 2008 Feb 20;3(2):e1613. doi: 10.1371/journal.pone.0001613 (PMC2238819; doi:10.1371/journal.pone.0001613)
Supplement: Table S3 — Non-interacting genes. Alleles tested for genetic interaction with a mblC overexpression phenotype that did not interact. (0.05 MB DOC) [file pone.0001613.s005.doc]

| **alleles** | **Gene** | **Description** |
| --- | --- | --- |
| *GS13048* | *CG10383* | similar to human serine active site containing 1 |
| *NP7046* | *CG10178* | toxin metabolism |
| *NP3393* | *Acp36DE* | extracelular protein, hormone activity, sperm competition, |
| *GS13720* | unknown | unknown |
| *GS94* | *Ntf-2r* | transport into nucleus |
| *GS10349* | unknown | unknown |
| *Df(1)w67c23, y[1] ;P{lacW}*  *l(2)k13805[k13805]* | l(2)k13805 | unknown |
| *GS9583* | unknown | unknown |
| *GS11118* | unknown | unknown |
| *GS11329* | *CG10373* | amino acid transporter activity |
| *GS7134, GS13361* | *Grip71* | gamma-tubulin binding |
| *Df(1)w67c23, y[1];P{lacW}*  *l(2)05847[k06526]* | *lethal(2)44DEa* | fatty acid metabolism |
| *NP 3378* | *Art2* | protein-arginine N-methyltransferase activity; |
| *Df(1)w67c23;P{lacW}*  *l(2)k08903[k08903]* | *Tps1* | disaccharide metabolism |
| *NP 3496* | *CG12677* | unknown |
| *GS9957* | unknown | unknown |
| *GS11509, GS9933* | *CG15439* | PHD Zn-finger, |
| *NP2314* | *CG15436* | Nucleic acid biding |
| *GS8126* | unknown | unknown |
| *Rnp^EP1082^* | *RnpS1* | mRNA catabolism, NMD |
| *slp1^2^* | *slp1* | transcription regulation,mesoderm formation |
| *bowl^11^* | *bowl* | regulation of transcription; cell proliferation |
| *Esg^35Ce-1^* | *esg* | fusion cell fate specification |
| *lolal^k02512^* | *lola-like* | regulation of transcription |
| *Ate1^k10809^* | *Ate1* | arginyltransferase activity |
| *stau^ry9^* | *stau* | RNA localization translation and splicing |
| *CG3249^EP1440^* | *Act5C* | cytoskeleton; |
| *Diap2^K02205^, Diap2^GMR.PH^* | *Diap2* | anti-apoptotic activity |
| *GS12289* | *bruno-2* | mRNA processing |
| *bru-3^EY8487^* | *bruno-3* | mRNA binding |
