# Supplementary material for: Drosophila Muscleblind Is Involved in troponin T Alternative Splicing and Apoptosis
Source: PLoS One. 2008 Feb 20;3(2):e1613. doi: 10.1371/journal.pone.0001613 (PMC2238819; doi:10.1371/journal.pone.0001613)
Supplement: Table S4 — Primer sequences used in this study. Names of primers used in the generation of GFP-tagged Bruno proteins include the first four characters of their cDNA names and the restriction site introduced. MblCK202I and MblC-PstI were used to perform site-directed mutagenesis on MblC-specific motif FKRP. TNTE2 and TNTE6 were used to amplify Drosophila troponin T transcripts. Rp49f and Rp49r amplify Rp49 mRNA as control for reverse transcriptase efficiency and RNA input. (0.04 MB DOC) [file pone.0001613.s006.doc]

| **Name** | **Sequence (5´to 3´)** | **Annealing T** |
| --- | --- | --- |
| **XhoILD29** | GGCCTCGAGATGTTCACCAGCCGCGCTTC |  |
| **XhoILD19** | GGCCTCGAGATGATGTTGCAATCCTTGAG |  |
| **XhoILD31** | GGCCTCGAGATGGTTCATATTATTGAATTG |  |
| **LD29EcoRI** | GGCGAATTCCAGTAGGGCTTCGAGTCCTTGG |  |
| **LD19BamHI** | GGCGGATCCTAAAAATTGCAAGTCGGAAAATGG |  |
| **LD31EcoRI** | GGCGAATTCCAATAGGGTCGACTGGCATC |  |
| **MblCK202I** | ACAACTTCCAATTCTCTGGCATGGTACCGTTCAUCCGTCC |  |
| **MlbC-PstI** | GGTACCGTCGACTGCGGAATTCCGTCTTGG |  |
| **RP49f** | ATGACCATCCGCCCAGCATAC | 65 |
| **RP49r** | ATGTGGCGGGTGCGCTTGTTC | 65 |
| **TNTE2** | CGACGATGAAGAGTACAC |  |
| **TNTE6** | CTCTGGATCGCCCTCTCC |  |
| **1938** | GCTGCAATAAACAAGTTCTGCTTT |  |
| **1956** | AGAATTGTAATACGACTCACTATAGGGC |  |
| **mblAd** | GAAGATCTTCGCTGAAATTGCGACCAAAAT | 55 |
| **mblAr** | CGGGATCCCGAATGAAAAACGAGCCTCCCT | 55 |
| **mblBd** | GAAGATCTTCACCATGTGTTTTTGCGTT | 55 |
| **mblBr** | CGGGATCCCGTAGGTGAAAGCCGAGAGCAT | 55 |
| **mblCd** | GAAGATCTTCTGTGTTTCCTGCGAAGTCC | 55 |
| **mblCr** | CGGGATCCCGGCATCGCTTGGGGAGTATT | 55 |
| **mblDd** | GAAGATCTTCCAACGACTTTGTGCTCTCCA | 55 |
| **mblDr** | CGGGATCCCGTCAACTGCTGGTTAAGCGTG | 55 |
